# Simvastatin Attenuates Hippocampal MMP-9 Expression in the Streptozotocin-Induced Cognitive Impairment

**DOI:** 10.29252/.23.4.262

**Published:** 2019-07

**Authors:** Soheila Adeli, Maryam Zahmatkesh, Mitra Ansari Dezfouli

**Affiliations:** 1 Department of Neuroscience and Addiction Studies, School of Advanced Technologies in Medicine, Tehran University of Medical Sciences, Tehran, Iran; 2 Electrophysiology Research Center, Neuroscience Institute, Tehran, Iran, Tehran University of Medical Sciences, Tehran, Iran; 3 Research Center for Cognitive and Behavioral Sciences, Tehran University of Medical Sciences, Tehran, Iran

**Keywords:** Alzheimer’s disease, Matrix metalloproteinase-9, Simvastatin

## Abstract

**Background:**

Matrix metalloproteinase-9 (MMP-9) expression has been implicated in molecular mechanisms of neurodegenerative disorders, and its abnormal level has been reported in Alzheimer’s disease (AD). Some protective mechanisms of statins against neurodegeneration might be mediated by the inhibition of MMP-9 expression. Here, we investigated the effect of simvastatin on the hippocampal MMP-9 expression in the context of AD.

**Methods:**

We examined the influence of three-week simvastatin (5 mg/kg) administration on hippocampal MMP-9 expression in a rat model of cognitive decline induced by streptozotocin (STZ). Spatial long-term memory and MMP-9 expression were assessed by Morris water maze (MWM) test and quantitative polymerase chain reaction, respectively.

**Results:**

The results showed a decline in the learning and memory in STZ group when compared with the control group. The MMP-9 up-regulated (1.41 ± 0.2 vs. 0.980 ± 0.02, *p* < 0.05), and cresyl violet staining showed hippocampal cell damage in STZ group compared with the control group. Simvastatin prevented the up-regulation of MMP-9 (1.05 ± 0.05 vs. 1.41 ± 0.2, *p* < 0.05), improved spatial memory impairment and attenuated hippocampal cell damage. Furthermore, we found a negative correlation (r = 0.77) between MMP-9 expression and cognitive function.

**Conclusion:**

Our findings suggest that the neuroprotective influence of simvastatin in battle to cognitive impairment is mediated in part by the modulation of MMP-9 expression. The reduction of MMP-9 expression in simvastatin-treated animals is in correlation with the improvement of cognitive functions. Understanding the protective mechanism of simvastatin will shed light on more efficient therapeutic modalities in AD.

## INTRODUCTION

Matrix metalloproteinases-9 (MMP-9), also known as gelatinase B, is a zinc-dependent endoprotease. Its expression has been implicated in molecular mechanisms of many pathological processes affecting the central nervous system[1]. Considerable researches have been conducted on the role of MMP-9 in neurodegenerative diseases[1,2]. In Alzheimer’s disease (AD), the higher levels of MMP-9 have been reported in the plasma[3,4] and hippocampus[5,6]. It has been shown that MMP-9 activity in the frontal and parietal cortex is greater in both AD and mild cognitive impairment compared to healthy subjects[7]. Increased hippocampal MMP-9 expression is involved in the development of cognitive impairmentinduced by beta-amyloid[8]. It has been demonstrated that laminin degradation by MMP-9 induces neuronal damage[9].

Clinical administration of statins is associated with the reduction of the AD incidence later in life[10]. Statins are HMG-CoA (3-hydroxy-3-methylglutaryl coenzyme A) reductase inhibitors commonly used for managing hypercholesterolemia. Simvastatin is a hydrophilic statin that can cross the blood brain barrier and has neuroprotective properties[11]. It exerts the strongantioxidant effect in the nervous system[12], attenuates glial activation[13] and reduces the expression of neuro-inflammatory mediators[14,15]. Moreover, simvastatinhas been shown to effectively protect blood brain barrierintegrity[16,17] and decrease the mRNA levels of MMP-9 in *in vitro* studies[18,19]. Various studies have focused on MMP-9 gene expression in neurodegenerative disorders [8,20]. It has been reported that some protective mechanisms of simvastatin may be mediated by the inhibition of MMP-9 gene expression[21]. There is no specific study centering the neuroprotective effect of simvastatin on memory performance in line with MMP-9 gene expression in the context of AD. In the present study, we evaluated the effect of oral simvastatin on hippocampal MMP-9 expression in a rat model of cognitive impairment induced by streptozotocin (STZ) infusion into the cerebral ventricles (ICV).

The ICV-STZ injection is a rat model of sporadic AD for preclinical testing of pharmacological therapies against AD[22,23]. The ICV-STZ injection decreases the cerebral glucose uptake, desensitizes brain insulin receptors, decreases the PI3K-AKT signaling activity and increases the activity of glycogen synthase kinase 3 beta[23,24]. These changes ultimately promote tau hyper-phosphorylation. Moreover, glucose hypo-metabolism initiates the process that ultimately results in Aβ aggregation[24] and induces memory impairment similar to sporadic Aβ pathology[25,26]. In the current study, we examined the effect of simvastatin administration on hippocampal MMP-9 expression in an animal model of cognitive impairment induced by intracerebroventricular (ICV) streptozotocin (STZ) administration.

## MATERIALS AND METHODS

### Animals

Male Albino Wistar rats, weighing 280–290 g, were used in all experimental procedures. Rats were kept under a controlled condition at 22 ± 2 °C and had free access to rat chow and water in their cages in a 12:12 hour light/dark cycle beginning with lights on at 7:00 am. All experiments were done in accordance with the guideline for the use of laboratory animals of the National Institutes of Health and approved by the Research and Ethics Committee of Tehran University of Medical Sciences, Tehran, Iran. All behavioral tests were performed between 9:00 am and 12:00 am.

### Brain surgery

For stereotaxic surgery, anesthesia was induced with intraperitoneal injection of ketamine and xylazine (60 mg/kg and 15 mg/kg, respectively; Alfasan, Woerden, Holland), and animals were positioned in the stereotaxic device (Stoelting Inc., USA). Stereotaxic coordinates for lateral ventricles were chosen (1.5 mm lateral to sagittal suture; -0.8 mm to Bergman; and 4 mm under the brain surface) according to the atlas of rat brain (Paxinos and Watson 2007). Guide cannulas were implanted bilaterally 1 mm above the lateral ventricles. The guide cannula was fixed with dental cement. During surgery, body temperature was kept at 36.5 ± 0.5 °C using a heating pad. After surgery, 50 mg/kg ampicillin was administered intramuscularly.

### Microinjection procedure

One week after surgery, general activity was assessed with the open field test. The exclusion parameters in the experiments were lack of normal locomotor activity, weight gain, and general health condition. STZ (1.5 mg/5 μL/side; Alexis, Lausen, Switzerland) or saline (5 μL/side) were infused on days 1 and 3 using a needle 1 mm longer than the guide cannula. The needle was attached to a polyethylene tube connecting to the 5-μl Hamilton microsyringe. Microinjections were performed slowly (0.4 μL/min), and the injection needle was left in place for a few additional minutes to avoid the backflow of injected materials. During the microinjections, the animals were free in their cage.

### Oral administration of simvastatin

Simvastatin (ab120505; Cambridge, UK) was dissolved in dimethyl sulfoxide (DMSO) 1%, and administered 5 mg/kg body weight through an oral gavage for three weeks after the first injection of STZ.

### Experimental groups

Rats were randomly assigned into four groups (eight rats per experimental group) as follows: (1) Saline + DMSO or vehicle group that received bilateral ICV infusion of saline (STZ vehicle) on days one and three and oral gavage of DMSO 1% (simvastatin vehicle) daily for three weeks; (2) STZ + DMSO group that received bilateral ICV infusion of STZ on days one and three and oral gavage of DMSO daily for three weeks. (3) STZ + Sim group that received bilateral ICV infusion of STZ and oral gavage of simvastatin daily for three weeks; (4) Saline + Sim group that received bilateral ICV infusion of saline on days one and three and oral gavage of simvastatin daily for three weeks.

### Spontaneous motor activity

Locomotor and exploration activity were evaluated with open field test as previously described[27]. The apparatus was made of a field measuring 80 × 80 cm surrounded by 40-cm-high walls. Animals were placed in the center of the open field device and allowed to move freely for five minutes. The incidence rate of line crosses and the number of rearing were measured to determine locomotor activity. The field was cleaned with 70% ethanol at the end of each session.

### Learning and memory assessment

All 32 rats were subjected to the behavioral tests. We evaluated spatial long-term memory using MWM test[28]. A black water tank (140 cm in diameter, 60 cm high, and depth of 30 cm) was filled with water (28 ± 1 °C). The water pool was placed in a silent room with several spatial cues. The water pool was divided into four equal parts. In the place navigation stages, the transparent escape platform (10 cm in diameter) was positioned at the midpoint of one quadrant. The rat could climb on the platform to escape from swimming. Each rat received four trials per day on days 17, 18, 19, and 20 after STZ injection. In each trial, the starting quadrant was selected randomly, and a maximum time of 60 s was given to rats to find the platform, and then they were allowed to stay on it for five s. On the first day, the rats were subjected to trials with visible platform. On the next three days, the platform was submerged below the water surface. Latency to escape from the water maze and the swimming distance to find the hidden platform were registered for each trial. Twenty-four hours after the last trial session, the platform was removed, and the probe test was done. The time spent and the distance traveled by the rat in the goal quadrant (where the platform was located during hidden platform training) were calculated. All data were recorded using video-tracking software (Radiab1 v2, Iran), which monitored the movements of rats above the center of the pool.

### RNA extraction, cDNA synthesis, and quantitative polymerase chain reaction (qPCR)

At the end of the experiment, the brains of all animals were removed. The hippocampus tissues of four animals in each group were used for RNA extraction and PCR analysis. Hippocampal total RNA was extracted using Trizol reagent (Zist Abzar Pajoohan Co., Iran). Purification of total RNA was evaluated using the Nanodrop 1000 Spectrophotometer (Thermofisher Scientific, USA). RNA quality was estimated by depicting 18S and 28S ribosomal RNA bands through electrophoresis. Then 500 ng of total RNA was reverse transcribed into cDNA using the PrimeScript RT kit (Thermofisher scientific) as stated by manufacturer’s protocol. Primers, RNA template, and nuclease free H_2_O mixture were kept warm at 65 °C for about 5 min. Subsequently, the solution was mixed with 2 μL of 5× PrimeScript buffer solution and incubated at 37 °C for 15 min and 85°C for 5 s and preserved at -20°C until further use. The resulting cDNA was used for quantitative measurement of gene expression levels of MMP-9 and GAPDH (glyceraldehyde 3-phosphate dehydrogenase) using the SYBR Premix Ex Taq II kit (Takara, Japan). The cycling conditions were 95 °C for 3 min, 35 cycles at 95 °C for 10 s, 59 °C for 10 s, and 72°C for 35 s. Real-time PCR was done in a real-time qPCR cycler instrument (Rotor-Gene Q, Qiagen), and the threshold cycle (Ct) was applied to quantify the mRNA level. Relative gene expressions were analyzed by 2^-ΔΔCt^ method, normalized to GAPDH, a housekeeping gene, and relative to the control group[29]. The primers used in the current experiment for MMP-9 and GAPDH are as follows: MMP-9: sense, ATCAGCCGGGAACG TATCTG; antisense, GTTGTGGAAACTCACACG CC; GAPDH: sense, GTTACCAGGGCTGCCTTCTC; antisense, GTTACCAGGGCTGCCTTCTC.

### Histological studies

After perfusion with PBS and ice-cold 4% paraformaldehyde, brain tissues of four animals in each group were fixed in 4% paraformaldehyde and then dehydrated in graded ethanol until became transparent and then embedded in paraffin. The blocks were cut coronally into serial five μm-thick sections. Selected sections from each block were treated with xylene and alcohol in different concentrations, rinsed in water and stained with 0.1% cresyl-violet acetate (Sigma-Aldrich, USA). The prepared slides were analyzed by the morphometric software (Optika Vision Pro, Italy). Surviving pyramidal neurons were quantified under 200× magnifications. Three fields of hippocampal CA1 regions were randomly selected. Intact neurons were counted, and the average number of neurons was calculated for analysis[30,31].

### Statistical analysis

Behavioral data were analyzed using repeated measures two-way ANOVA and one-way ANOVA. Comparison between the groups in the molecular and histological studies was made by one-way ANOVA. The post-hoc test was Tukey. Pearson correlation test was applied to investigate the association between gene expression and cognition. SPSS 16.0 software was used for statistical analysis. In all statistical analyses, *p* values less than 0.05 was considered as significant.

## RESULTS

### Simvastatin showed no effect on locomotor activity in the open field test

We performed open field test to evaluate locomotor activity after the recovery of stereotaxic surgery. The number of line crosses and rearing behavior during five min was recorded in each session. As presented in Table 1, there was no significant change between groups in the number of line crosses [F(3, 28) = 0.57]. Moreover, no significant difference was detected in the number of rearing among different groups [F(3, 28) = 0.96].

**Table 1:** Data of general activity in different groups (n = 8 in each group)

Groups	Open field test	

	Number of line crosses	Frequency of rearing
Saline + DMSO	46.7 ± 2.57	7.25 ± 0.75
Saline + Sim	46.75 ± 4.51	6.37 ± 0.77
STZ + DMSO	52.50 ± 5.10	8.25 ± 1.10
STZ + Sim	46.50 ± 2.38	6.62 ± 0.59

Values are expressed as mean ± SEM. No significant difference was seen between the groups.

### Simvastatin improved learning and memory in the MWM test

**Fig. 1:**
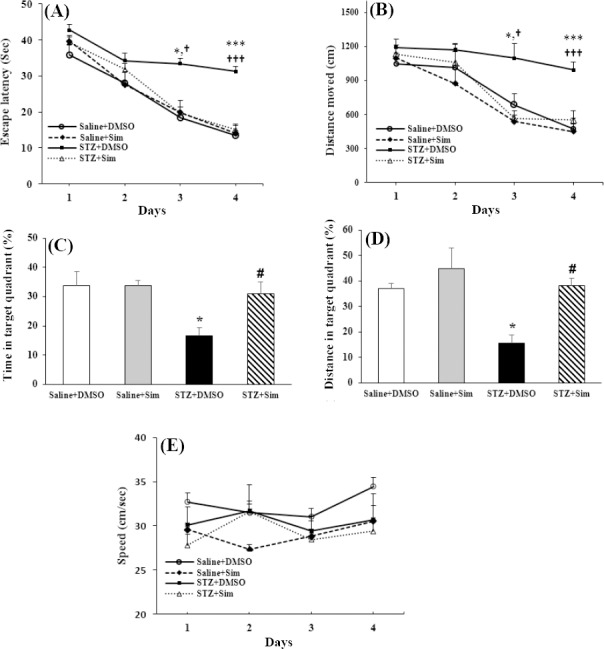
Effects of simvastatin (Sim) administration on learning and memory function of rats in Morris water maze test (n = 8 in each group). During place navigation stages on days 17-20 following STZ injection, time taken for the rats to find the platform (a) and total distance traveled (b) in each trial were measured. Percentage of time (c) and distance (d) spent in the goal quadrant (platform area) during probe test is shown in the Figure. There were significant differences between STZ + Sim and STZ + DMSO in distance moved and escape latency during days three and four of place navigation stages. Swimming speed did not change significantly in different groups during four days of place navigation (e). Values are expressed as mean ± SEM. ^*^p < 0.05 and *^**^p < 0.001 compared with Saline + DMSO group; ^#^*p* < 0.05 compared with STZ + DMSO group; ^†^*p* < 0.05 and ^†††^*p* < 0.001 compared with STZ + Sim group.

The MWM test was performed to assess the hippocampal-dependent learning process and spatial reference memory. Animals with cognitive impairment spent more time and traveled more distance to find the platform during the trials of MWM test. The total distance moved and escape latency are shown in Figure 1. We found a significant difference between the treated groups in escape latency [F(3, 28) = 202.97, *p* < 0.001, Fig. 1A] and distance traveled [F(3, 28) = 89.3, *p* < 0.001, Fig. 1B]. The Saline + DMSO group showed a progressive reduction in escape latency over successive trials, similar to the Saline + Sim group. The STZ + DMSO group compared with Saline + DMSO and Saline + Sim groups indicated significantly prolonged escape latency during trials on day three (18.4 ± 4.9 or 19.9 ± 2.3 vs. 33.4 ± 1.4 sec, F(3, 28) = 4.52, *p* < 0.05) and day four (13.5 ± 2.9 or 14.0 ± 0.7 vs. 31.3 ± 1.3 sec, F(3, 28) = 21.72, *p* < 0.001). The mean escape latency to find the platform significantly improved in the STZ + Sim group compared with STZ + DMSO on both day three (19.7 ± 2.3 vs. 33.4 ± 1.4 sec, *p* < 0.05) and day four (15.0 ± 1.6 vs. 31.3 ± 1.3 sec, *p* < 0.001). The total distance traveled was significantly longer for the STZ + DMSO group compared with the Saline + DMSO and Saline + Sim on day three (684.2 ± 96 or 537.6 ± 94.0 vs. 1097.2 ± 127.2 cm, *p* < 0.05) and day four (476.0 ± 92.1or 448.9 ± 66.6 vs. 993.5 ± 70.4 cm, *p* < 0.001). The distance traveled significantly decreased in the STZ + Sim group in comparison to the STZ + DMSO group on both day three (566.9 ± 25.9 vs. 1097.2 ± 127.2 cm, *p* < 0.05) and day four (551.6 ± 84.6 vs. 993.5 ± 70.4 cm, *p* < 0.001). Swimming velocity did not change significantly in different groups during four days of place navigation (F(3, 28) = 1.65, *p* > 0.05, Fig. 1E).

In the probe test, our data showed significant difference between the groups in the percentage of time spent [F(3, 28) = 5.3, *p* < 0.05, Fig. 1C]) and distance traveled [F(3, 28) = 6.9, *p* < 0.001, Fig. 1D] in the goal quadrant. The percentage of time exploring in the goal quadrant decreased in the STZ + DMSO compared with the Saline + DMSO and Saline + Sim groups (16.7 ± 2.8 vs. 33.82 ± 4.8 and 33.80 ± 1.6 *p* < 0.05). The percentage of distance traveled in the goal quadrant in the Saline + DMSO group was similar to that of the Saline + Sim group, while it significantly decreased in the STZ + DMSO group in comparison with Saline + DMSO and Saline+Sim groups (37.1 ± 1.8 and 44.7 ± 8.3 vs. 15.7 ± 2.9, *p* < 0.05). However, the percentage time of exploration in the goal quadrant in the probe test significantly raised in the STZ + Sim group when compared to the STZ + DMSO group (31.0 ± 3.9 vs. 16.7 ± 2.8, *p* < 0.05). The percentage of distance significantly elevated in the STZ + Sim group compared with the STZ + DMSO group (38.2 ± 3.1 vs. 15.7 ± 2.9, *p* < 0.05).

### Simvastatin reduced MMP-9 gene expression in hippocampus

**Fig 2:**
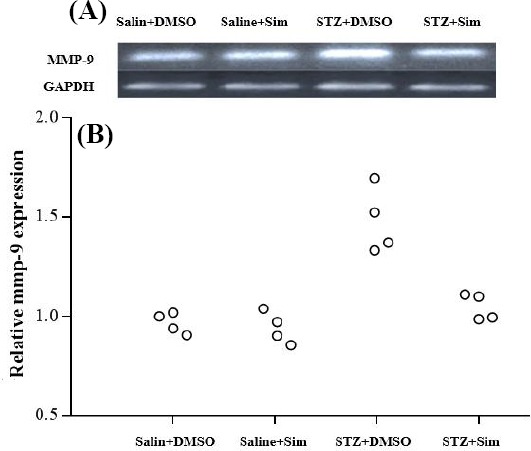
Relative MMP-9 expression in hippocampus tissue of different groups. (a) The gel electrophoresis of PCR products of MMP-9 and GAPDH in different groups; (b) the relative expression of MMP-9 gene in different groups. The hippocampus tissues were isolated from four animals in each group, and each hippocampus was assessed three replicate times. The circles show the data of each rat in a group.

Hippocampal MMP-9 gene expression level was evaluated using quantitative real-time PCR. The hippocampus tissues of four animals in each group were isolated for MMP-9 gene expression, and the PCR results of each animal were repeated for three consecutive times. As depicted in Figure 2, no significant difference was detected in MMP-9 expression between the Saline + DMSO and Saline + Sim groups. However, MMP-9 expression was upregulated in the STZ + DMSO group compared with Saline + DMSO and Saline + Sim groups (1.41 ± 0.02 vs. 0.987 ± 0.01 and 0.980 ± 0.02, *p* < 0.05). Simvastatin administration significantly prevented the rise of MMP-9 expression in the STZ + Sim group relative to STZ + DMSO group (1.05 ± 0.05 vs. 1.41 ± 0.2, *p* < 0.05).

### A negative correlation was found between hippocampal MMP-9 expression and cognitive indices

**Fig. 3:**
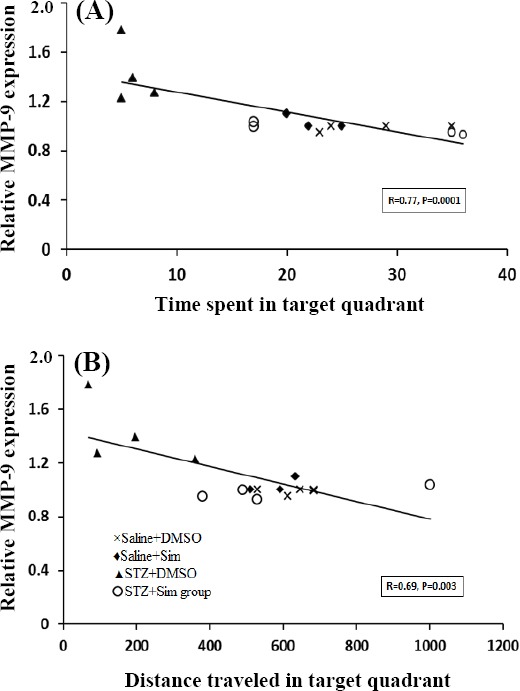
Correlation analysis between MMP-9 relative expression and time spent in the goal quadrant (a) and total distance traveled (b) in MWM, as indexes of cognitive function.

As presented in Figure 3, Pearson correlation analysis demonstrated a significant negative relationship between MMP-9 gene expression in hippocampus and time spent (r = 0.77, Fig. 3A) and distance moved (r = 0.69, Fig. 3B) in the goal quadrant (as cognitive indices) during probe test in the MWM test.

### Simvastatin improved the morphological features of hippocampal neurons

**Fig. 4:**
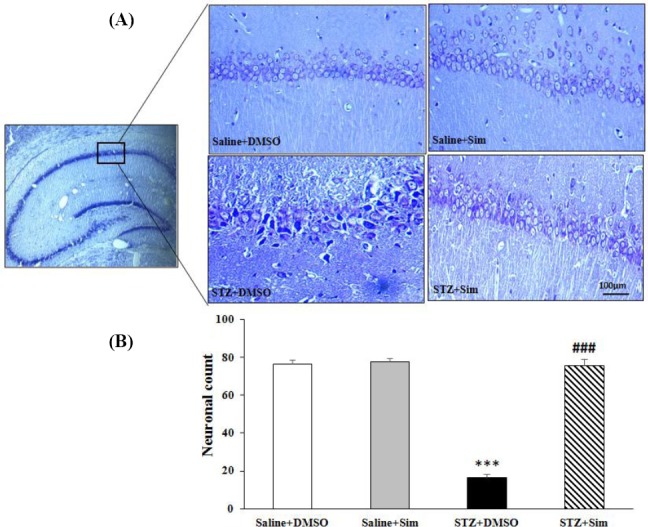
Alteration of the CA1 hippocampal pyramidal cells morphology in different groups. Following the completion of the experiment, four hippocampal tissues from each group (n = 4) were processed for cresyl violet staining. (a) Intact morphology and regular arrangement in Saline + DMSO, Saline + Sim, and STZ + Sim groups. In STZ + DMSO group, cell bodies were shrunken with unpacked arrangement in hippocampal tissue. (b) The neuronal count in the hippocampal CA1 regions in slide fields in different groups. Values are expressed as mean ± SEM. ^***^p < 0.001 compared with Saline + DMSO; ^###^*p* < 0.001 compared with STZ + DMSO group.

Morphology and cellular arrangement of pyramidal neurons in hippocampus tissue were monitored by cresyl violet staining. Pyramidal neurons in the hippocampus tissues of the Saline + DMSO, Saline + Sim, and Saline + Sim groups had normal morphology with densely packed arrangement. Damaged cells with shrunk cell bodies were noted in the hippocampus sections of the Saline + STZ group (Fig. 4A). There were significant changes in neuron count on different treatment samples between the groups (F (3, 12) = 156.1, *p* < 0.001). No significant difference was seen in hippocampal cell count in the Saline + DMSO and Saline + Sim groups. The neuron count in the STZ + DMSO group significantly reduced compared with the Saline + DMSO and Saline + Sim groups (76.5 ± 2.1 and 77.7 ± 3.5 vs 16.5 ± 1.5, *p* < 0.001). The neuron count in hippocampal sections increased in the STZ + Sim group relative to the STZ + DMSO group, as shown in Figure 4B (75.5 ± 1.8 vs. 16.5 ± 1.5, *p* < 0.001). These data showed the neuroprotective effect of simvastatin on hippocampal pyramidal cells against neural damage induced by ICV-STZ injection.

## DISCUSSION

In the present study, a significant cognitive decline, recognized by prolonged mean escape latency, was detected in the STZ group when compared to the control group. This observation is in line with previous findings regarding spatial memory decline in this model[32,33]. The presence of shrunk cell bodies in hippocampus sections of the STZ group confirmed the induction of injury[34]. The present results showed that hippocampal MMP-9 up-regulated in the rat model of cognitive impairment induced by intracerebro-ventricular injection of STZ. Our results are in agreement with a previous finding that demonstrated MMP-9 involvement in the pathogenesis of brain injuries and cognitive disorders[35]. Elevated levels of MMP-9 were localized in the cytoplasm of neurons, neurofibrillary tangles, senile plaques, and vascular walls in postmortem sections of the parietal lobe and hippocampus from AD patients[5]. The greater proteolytic activity of MMP-9 in the postmortem frontal and parietal cortical tissues of AD patients has been observed[7,36]. MMP-9 is expressed in the various regions of brain including the hippocampus[37] and localized and released from neurons, astrocytes, and microglia[38]. MMP-9 overexpression activates cytokines and enhances neuro-inflammation and celldeath[39]. Elevated level of MMP-9 has been reported in the transgenic mouse AD model[40]. Increased levels of MMP-9 expression have also been reported in the hippocampus and cerebral cortex of AD-affected patients[5,6]. Overexpression of MMP-9 is associated with disturbed hippocampal long-term potentiation[41]. Therefore, the prolonged mean escape latency in the present study may in part be related to the overexpression of MMP-9 in the STZ group. One study, which performed a quantitative gel zymography, has found no change in plasma MMP-9 activity in AD patients[42]. This observation may partly be due to the discrepancies between the enzymatic activity and gene expression of MMP-9. Moreover, in this study, we found a negative correlation between MMP-9 expression and cognitive function in rats from all groups, which implies that MMP-9 level may contribute to cognition impairment in AD pathology. A number of studies have focused on MMP-9 gene expression in neurodegenerative disorders [8,20].

Recently, statins have been noticed as effective neuroprotective molecules[43]. In our study, simvastatin prevented the rise of MMP-9 expression in the STZ group, decreased neural damage in hippocampal morphology and eventually improved spatial long term memory.

In the current work, administration of simvastatin for three weeks enhanced learning and memory in the STZ + Sim group. Many clinical studies have reported that statins have a significant effect on memory in patients[44-46], whereas some others have failed to demonstrate this beneficial effect[47,48]. This controversy in clinical reports may be, to some extent, due to the variations in the study design, dose and duration of statin administration, and methods of cognition examination[49].

As presented in the current study, simvastatin improved neural density in hippocampal tissue in the STZ + Sim group. Another study has shown suppressed neural death in the hippocampus by simvastatin[49]. Simvastatin modulates MMP-9 expression and reduces neuro-inflammation and apoptosis[15,50]. It has been reported that simvastatin hinders oxidative stress through increasing the expression of hippocampal antioxidant enzymes[51]. It has also been reported that simvastatin improves memory impairment after cerebrovascular damage[52,53], prevents oxidative damages, and enhances long-term potentiation[25,54]. These protective actions may explain the enhancement in hippocampal cell count and neuronal density in the ICV-STZ injected rats.

Oral administration of simvastatin, 5 mg/kg body weight for three weeks after STZ infusion, prevented the up-regulation of MMP-9 gene expression, reduced neuronal damage in hippocampal tissue and subsequently improved cognitive impairment. We also observed negative correlation between MMP-9 gene expression and spatial memory capability. Our findings suggest that the modulation of MMP-9 gene expression may be a neuroprotective scheme of simvastatin in battle with the cognitive decline in AD.
